# Influenza A virus infection dysregulates the expression of microRNA-22 and its targets; CD147 and HDAC4, in epithelium of asthmatics

**DOI:** 10.1186/s12931-018-0851-7

**Published:** 2018-08-02

**Authors:** Fatemeh Moheimani, Jorinke Koops, Teresa Williams, Andrew T. Reid, Philip M. Hansbro, Peter A. Wark, Darryl A. Knight

**Affiliations:** 1 School of Biomedical Sciences and Pharmacy, Faculty of Health and Medicine, The University of Newcastle, HMRI building, Callaghan, NSW 2308 Australia; 2 Priority Research Centre for Healthy Lungs, Hunter Medical Research Institute, The University of Newcastle, Newcastle, NSW Australia; 3 Department of Molecular Pharmacology, University of Groningen, Groningen, Netherlands; 4 Department of Biochemistry and Microbiology, University of Victoria, Victoria, Canada; 5 Department of Respiratory and Sleep Medicine, John Hunter Hospital, Newcastle, NSW Australia; 6 Department of Anesthesiology, Pharmacology and Therapeutics, University of British Columbia, Vancouver, Canada

**Keywords:** Severe asthma, Epithelial cells, microRNA, Influenza A virus, Airway remodeling

## Abstract

**Background:**

Specific microRNAs (miRNAs) play essential roles in airway remodeling in asthma. Infection with influenza A virus (IAV) may also magnify pre-existing airway remodeling leading to asthma exacerbation. However, these events remain to be fully defined. We investigated the expression of miRNAs with diverse functions including proliferation (miR-20a), differentiation (miR-22) or innate/adaptive immune responses (miR-132) in primary bronchial epithelial cells (pBECs) of asthmatics following infection with the H1N1 strain of IAV.

**Methods:**

pBECs from subjects (*n* = 5) with severe asthma and non-asthmatics were cultured as submerged monolayers or at the air-liquid-interface (ALI) conditions and incubated with IAV H1N1 (MOI 5) for up to 24 h. Isolated miRNAs were subjected to Taqman miRNAs assays. We confirmed miRNA targets using a specific mimic and antagomir. Taqman mRNAs assays and immunoblotting were used to assess expression of target genes and proteins, respectively.

**Results:**

At baseline, these miRNAs were expressed at the same level in pBECs of asthmatics and non-asthmatics. After 24 h of infection, miR-22 expression increased significantly which was associated with the suppression of CD147 mRNA and HDAC4 mRNA and protein expression in pBECs from non-asthmatics, cultured in ALI. In contrast, miR-22 remained unchanged while CD147 expression increased and HDAC4 remained unaffected in cells from asthmatics. IAV H1N1 mediated increases in *SP1* and *c-Myc* transcription factors may underpin the induction of CD147 in asthmatics.

**Conclusion:**

The different profile of miR-22 expression in differentiated epithelial cells from non-asthmatics may indicate a self-defense mechanism against aberrant epithelial responses through suppressing CD147 and HDAC4, which is compromised in epithelial cells of asthmatics.

**Electronic supplementary material:**

The online version of this article (10.1186/s12931-018-0851-7) contains supplementary material, which is available to authorized users.

## Background

Airway remodelling in asthmatics is defined as structural abnormalities including, epithelial integrity loss, basement membrane thickening, and goblet cell and submucosal gland enlargement [1]. Airway remodelling may predispose asthmatics to exacerbations [2]. The airway epithelium consists of multiple distinct cell types; ciliated columnar, goblet, side population, serous and basal cells [3]. Basal cells are capable of self-renewal and differentiation into mucus-secreting and ciliated cells [4, 5]. The epithelium of asthmatics displays a greater proportion of basal and fewer ciliated cells [6], suggesting dysregulated differentiation. Particularly in severe asthma, increased epithelial proliferation contributes to thickened epithelium and *lamina reticularis* and hence airway narrowing [7, 8]. Dysregulated epithelial differentiation therefore plays an important role in the remodelling process in asthma. These abnormalities are also associated with functional aberrations including deficient innate immune responses [3, 9, 10]. The innate immune function of the epithelium is essential for defence against inhaled pathogens such as viruses [3, 11–13]. The differentiation state of the airway epithelium is also important for innate immunity through the compartmentalization of receptors and mediator production [4]. Consequently, structural and functional abnormalities in the epithelium may contribute to increased susceptibility of asthmatics to noxious environmental stimuli, including respiratory viruses (e.g. influenza A virus [IAV]).

The IAV H1N1 causes significant morbidity and mortality in annual seasonal epidemics [14]. This virus damages the epithelium [15] and triggers inflammation and cell signalling events resulting in additional airway remodelling and potentially exacerbations of asthma [14].

microRNAs (miRNAs) are small non-coding RNAs which regulate the expression of up to 60% of human genes [16]. Further, changes in specific miRNAs during human airway epithelial cell differentiation regulates gene and protein expression important for differentiation [17]. miRNAs are hence crucial in most biological and pathological processes [18, 19], including severe asthma [20]. Some miRNAs, such as miR-20a from the miR-17-92 cluster, promote the proliferation of lung epithelial progenitor cells [21]. Whereas, others such as miR-22, are differentiation specific and suppress different genes responsible for cell proliferation [10, 22–24]. Several miRNAs are also associated with the regulation of innate and adaptive immunity, including miR-132 [25, 26]. Consequently, miRNAs may play a major role in phenotypic and functional abnormalities of airway epithelial cells. IAV H1N1 infection is also reported to dysregulate the expression of some miRNAs in human lung epithelial cells, affecting immune responses [27, 28].

In this study, we hypothesized that the expression of miRNAs responsible for proliferation, miR-20a, are elevated, whereas miRNAs associated with differentiation, miR-22, are down-regulated in airway epithelial cells of asthmatics. These defects may form the link between abnormal airway epithelial cell differentiation and remodelling. In addition, IAV H1N1 infection may further dysregulate abnormalities in these miRNAs and hence their targets in the airway epithelial cells of asthmatics. Thus, we assessed the expression and role of these miRNAs in the context of airway remodelling in primary bronchoepithelial cells (pBECs) obtained from asthmatics, cultured as monolayers or differentiated ALI conditions at baseline level and after IAV H1N1 infection.

## Methods

### Cell culture

This study was approved by the Human Research Ethics Committee of The University of Newcastle. Human pBECs were obtained from non-asthmatics and adults with severe or difficult to treat asthma based on international ERS/ATS guidelines [29] by endobronchial brushing during fibre-optic bronchoscopy [30]. Donors had no history of smoking. Non-asthmatics had no lung disease and had normal lung function. See Table 1 for patients’ demographics. All subjects gave written consent. Experiments were conducted on cells at passage 2. pBECs were cultured as submerged monolayers or at ALI as previously described [31]. Experiments were carried out on day 23–25 after raising cell culture to ALI (Additional file 1: Figure S1). Minimally-immortalized BECs (HBEC6-KT) were generously provided by Dr. John Minna [32] and maintained in Keratinocyte Serum-Free Media (KSFM; Invitrogen) with growth supplements and antibiotics as described previously [33]. Madin-Darby canine kidney (MDCK) cells (American Type Culture Collection, USA) were maintained in Dulbecco’s modified Eagle’s media with 5% fetal bovine serum [34].

**Table 1:** Donor demographics

Characteristics	Healthy controls	Asthmatics	*P* Value
n	14	14	NA
Sex (M/F)	3/11	6/8	0.2
Mean age (SD), yr	58.1 (10.3)	54.7 (15.0)	0.5
Mean FEV1% predicted (SD)	100.1 (14.5)	60.7 (23.4)	< 0.0001
FEV1:FVC ratio (SD)	80.4 (3.5)	59.3 (12.4)	< 0.0001
Inhaled corticosteroid, % treated	0	100	NA
Atopy	0	7	< 0.05

Definition of abbreviations: NA, not applicable. The statistical analysis used for this table was using unpaired T-test with Welch’s correction

### IAV H1N1

Human IAV A/Auckland/1/2009 (H1N1) strain was obtained from the WHO Collaborating Centre for Reference and Research on Influenza (Victoria, Australia). IAV H1N1 was propagated and titrated in MDCK cells [12, 13, 35]. The titer of virus stock was determined by plaque assay on MDCK cells [34]. Ultra-violet (UV) inactivation of live viruses was achieved by placing live viruses directly under UV lamp (254 nm) for 2 h [12, 13, 35]. Successful inactivation was confirmed by plaque assays.

### Cell viability

IAV H1N1 was diluted in serum free KSFM media and added to HBEC6-KT cultured as monolater at a multiplicity of infection (MOI) of 0.5, 1, 5, 10 and 20. After 1 h of incubation, inocula were removed and replaced with serum-free media for 24 h. Cell viability was assessed by lactate dehydrogenase release [36] at 24 h post infection.

### IAV H1N1 infection

IAV H1N1 was diluted in the appropriate serum free media and added to pBECs cultured at monolayer or to apical surface of ALI culture, at MOI of 5 [12, 13, 35]. After 1 h or 6 h of incubation of cells cultured under monolayers or ALI conditions, respectively, the inocula were removed and replaced with serum-free media. At 0, 1, 4, 6 and 24 h post inoculation of pBECs cultured as monolayers or 0, 6, 8 and 24 h post infection of pBECs cultured at ALI, miRNAs and mRNAs were isolated using RNAeasy Mini Kit (Qiagen, USA) using 100% instead of 70% ethanol, proteins were lysed [33], and media were collected and stored at − 80 °C. Viral infection was confirmed by plaque assay using MDCK cells [34].

### Cell transfection

HBEC6-KT cells or pBECs were seeded at 1 × 10^5^ cell per well in 24-well plates 24 h before transfection. Cells were transfected, as described previously [37], with miR-22 mimic, miRNA mimic negative control (mirVana), miR-22 inhibitor, or miRNA inhibitor negative control (mirVana) at 5 nM total concentration using Lipofectamine® RNAiMAX Transfection Reagent (invitrogen) in Opti-MEM Medium (Gibco).

### RT-qPCR

miRNA (100 ng) or mRNA (200 ng) were reverse transcribed to cDNA, using Taqman MicroRNA Reverse Transcription reagent or High Capacity cDNA Reverse Transcription Kit (Applied Biosystems). Predeveloped primer/probe sets were purchased from Applied Biosystems (TaqMan miRNA assays or TaqMan gene assays). The quantitative fluorogenic amplification of cDNA (RT-qPCR) was performed using an ABI 7500 Real Time PCR (Applied Biosystems) or Eppendorf RealPlex (Eppendorf) instruments, respectively. RNU44 (based on manufacturer’s recommendations (Applied Biosystem) and previous reports [38, 39]) or ribosomal RNA (18S) [40, 41] was used as the reference miRNA or gene, respectively. The cycle threshold (Ct) value obtained was normalized to that of the RNU44 or 18S gene (∆Ct). Data were expressed as 2^-∆∆Ct^ [42] where ∆∆Ct was calculated from subtracting individual ∆Ct from ∆Ct of non-asthmatic media control at baseline.

### Immunoblotting

pBECs were lysed in RIPA buffer, and all proteins were standardized to 5 μg and were resolved by SDS-PAGE, and transferred onto nitrocellulose membranes for detection of CD147, histone deacetylase (HDAC) 4, MMP-9, c-Myc and SP1 (Abcam, UK), in cell lysates. Glyceraldehyde 3-phosphate dehydrogenase (GAPDH) was detected as a loading control for proteins in cell lysates [12, 13]. Protein estimation was determined by densitometry, using Image lab software (version 4.1), and values were expressed as protein/GAPDH ratio and normalized to media controls at baseline from pBEC of non-asthmatics. All antibodies used in this study are commercially available and have been validated by the source company.

### Gelatin zymography

Activity of MMP-9 released from pBECs into basal media of cells cultured at ALI was assessed by gelatin zymography, as previously described [43], with the following modifications. Four times nonreducing loading buffer was used and samples (20 μg) were resolved in 10% ready zymogram gels (BioRad). Gels were electrophoresed at 125 V for 90 min at 4 °C. The developing buffer consisted of 50 mM Tris, 200 nM NaCl, 5 mM CaCl_2_ (anhydrous), 0.02% Brij-35 and adjusted to pH 7.5; the staining buffer consisted of 40% methanol, 10% acetic acid and 0.5% Coomassie Blue R-250, and; the destaining solution consisted of 40% methanol and 10% acetic acid. Gelatin-degrading MMP-9 enzyme was identified as clear zones of lysis against a blue background. Molecular masses of gelatinolytic bands were estimated using prestained molecular mass markers; precision plus protein standard (BioRad). Activities in the gel were determined by densitometry, using Image lab software (version 4.1).

### Statistical analysis

Data were expressed as mean ± standard error of mean (SEM). Nonparametric test was used for pair-wise comparisons, and Kruskal Wallis nonparametric multiple comparisons test was used for comparison of group data when non-normally distributed. A two-way analysis of variance with Bonferroni post-test was used for comparison of group data when normally distributed. A *p*-value of ≤ 0.05 was considered significant.

## Results

### Morphological and physical properties of monolayer and ALI cultures of pBECs from non-asthmatics and asthmatics

In order to assess and compare the expression of miRNAs in homogenous populations of pBECs [44], we cultured cells in monolayer culture (Additional file 1: Figure S1A-B) as well as ALI culture, where cells differentiate and form cilia and secrete mucus which is more representative of the pseudostratified structure (Additional file 1: Figure S1C-D). We observed no morphological difference between pBECs from non-asthmatics and asthmatics in monolayer culture. pBECs from both non-asthmatics and asthmatics formed differentiated, multilayered ALI cultures, also with no obvious histological differences between them (Additional file 1: Figure S1C-D). In cells cultured at ALI, the formation of junctional complexes was indirectly confirmed by measurement of transepithelial electrical resistance (TEER) every 7 days, using World Precision Instrument Inc. (Sarasota, FL, USA) [45]. We observed increases in TEER during the differentiation stage of pBECs, which reached a statistically significant level on days 14, 21 and 23–25 (pre-infection) in non-asthmatics (Additional file 1: Figure S1E), and days 21 and 23–25 in asthmatics (Additional file 1: Figure S1F). However, no difference was observed between the TEER of pBECs from asthmatics compared with no-asthmatics (Additional file 1: Figure S1E-F).

### Expression of candidate miRNAs in pBECs

We initially confirmed that H1N1 IAV (MOIs 0.5, 1, 5 and 10) infection for 24 h had no effect on cell viability compared with cells incubated with UV-inactivated IAV H1N1 or media, whereas MOI 20 caused a significant cell death (Additional file 1: Figure S2). To investigate the effect of IAV H1N1 on expression of candidate miRNAs, pBECs cultured in monolayers or at ALI culture were infected (MOI 5 [12, 13, 35] for 1 or 6 h, respectively and allowed to recover over 24 h). Changes in appearance of MDCK cells, including enlarged nuclei and cellular granulation [46] confirm infection in cells cultured in monolayers from both groups (Additional file 1: Figure S3). Further, our data show that pBECs from non-asthmatics cultured at ALI had the same viral titers as cells from asthmatics 24 h post infection (Additional file 1: Figure S4 A-B) [34] and indicate that epithelial cells of non-asthmatics and asthmatics are infected to the same level.

We further confirmed that RNU44 is expressed in pBECs, is not affected by disease state (asthma) or H1N1 infection and hence is an appropriate endogenous control for miRNAs (Additional file 1: Figure S5). We also showed that pBECs incubated with UV-inactivated IAV H1N1 expressed similar levels of miR-20a, − 132 and − 22 as media controls at corresponding time points and this was consistent in both culture conditions (Additional file 1: Figure S6). Thus, our subsequent data from cells infected with IAV H1N1 were compared with corresponding data from cells cultured with media.

**Fig. 1:**
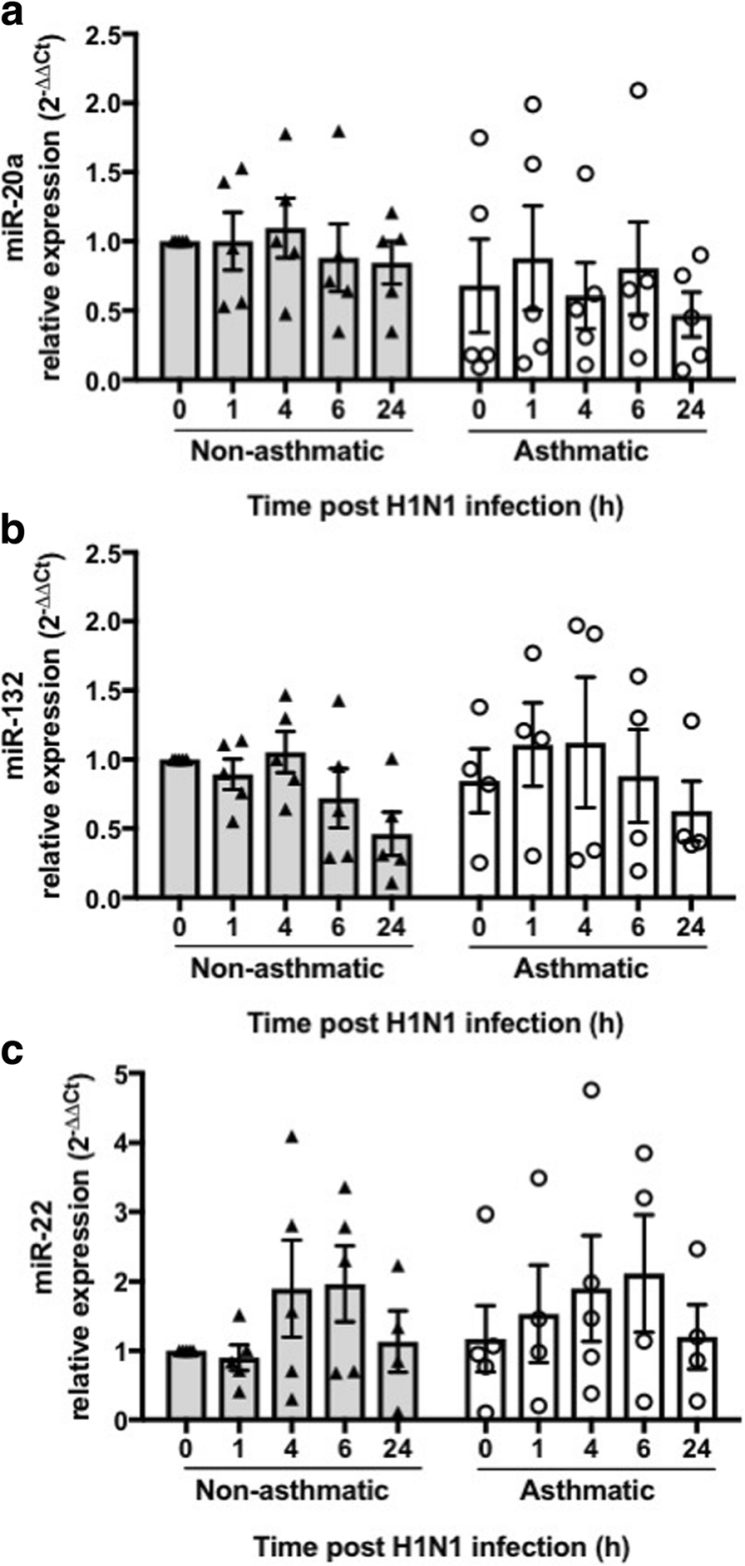
Effect of IAV H1N1 infection on miRNA expression in pBECs from non-asthmatics and asthmatics cultured as monolayers. Cells were infected with IAV H1N1 (MOI 5) and the expression of candidate miRNAs were assessed at different time points (0, 1, 4, 6 and 24 h). **a** miR-20a, (**b**) miR-132, and (**c**) miR-22, were expressed at similar levels in non-asthmatics and asthmatic cells, at baseline (0 h) or after IAV H1N1 infection (1–24 h), using Kruskal Wallis multiple comparisons test, *N* = 5. The cycle threshold (Ct) value was normalized to that of the RNU44 (∆Ct). Data are presented relative to corresponding non-asthmatic levels at baseline

We initially observed similar expression of miR-20a, − 132, and − 22 in pBECs from asthmatic and non-asthmatics cultured as monolayers in the absence of any stimulus at baseline (0 h) (Fig. 1). Thereafter, we infected pBECs cultured as monolayers with IAV H1N1 (MOI 5) and assessed the expression of candidate miRNAs at different time points (1, 4, 6 and 24 h). Our data show that expressions of these miRNAs were not affected to statistically significant levels by IAV H1N1 infection in cells from either non-asthmatics or asthmatics, compared with relevant control. The pattern of expression of these miRNAs was also similar in both group (Fig. 1).

**Fig. 2:**
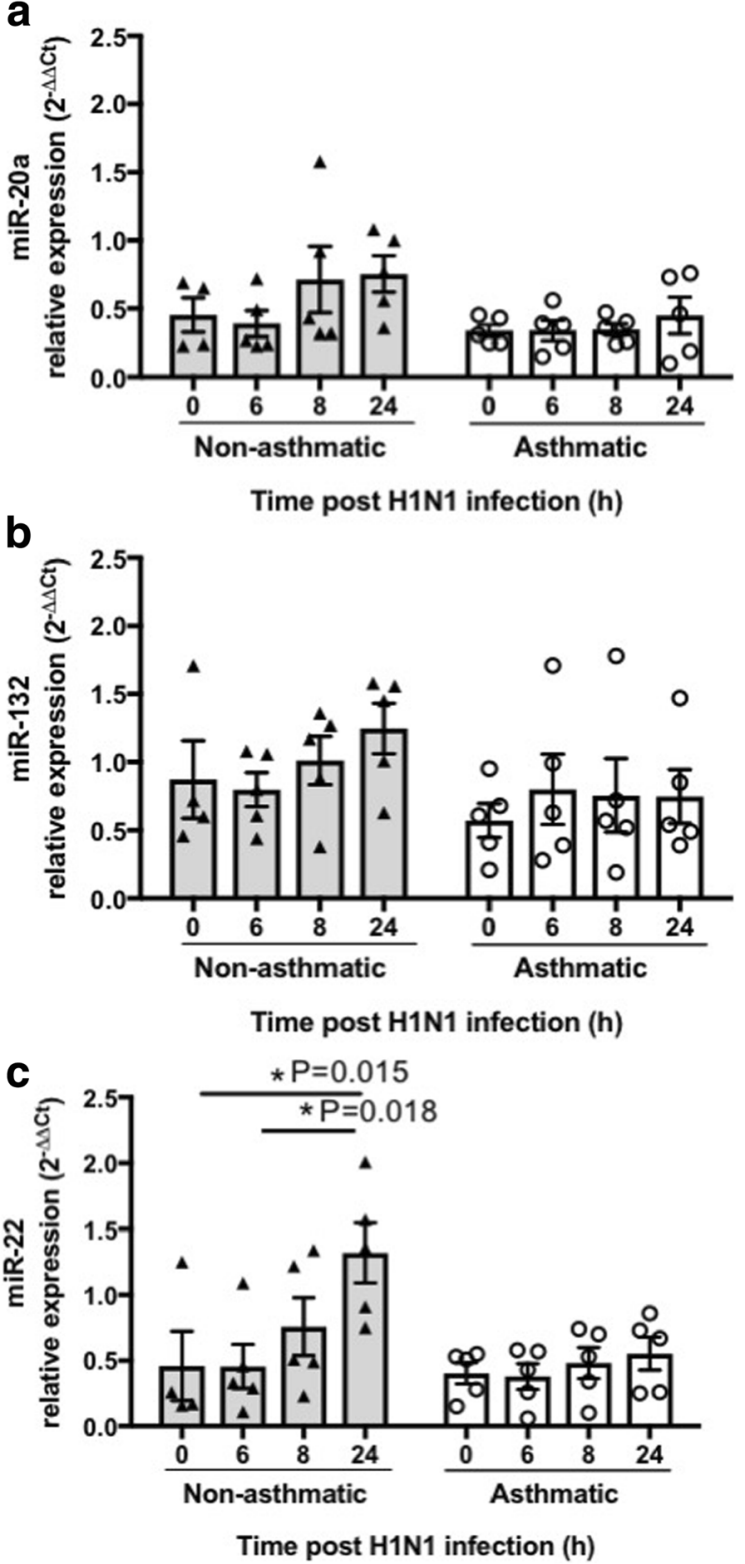
Effect of IAV H1N1 infection on miRNA expression in pBECs from non-asthmatics and asthmatics cultured at ALI. Cells were infected with IAV H1N1 (MOI 5) and the expression of candidate miRNAs were assessed at different time points (0, 6, 8 and 24 h). **a** miR-20a, (**b**) miR-132, and (**c**) miR-22, were expressed at similar levels in non-asthmatics and asthmatic cells, at baseline (0 h). H1N1 infection had no effect on the expression of (**a**) miR-20a, and (**b**) miR-132, whereas (**c**) miR-22 expression increased after infection in cells form non-asthmatics but not in asthmatics, *P ≤ 0.05, using the Kruskal Wallis multiple comparisons test, *N* = 5. The cycle threshold (Ct) value was normalized to that of the RNU44 (∆Ct). Data are presented relative to corresponding non-asthmatic levels at baseline

The baseline expression (0 h) of these miRNAs were also similar in pBECs from asthmatics and non-asthmatics when cultured at ALI (Fig. 2). IAV H1N1 infection did not alter the expression of miR-20a, and − 132 (Fig. 2a-b), in ALI cultures. miR-22 expression however increased over time in pBECs from non-asthmatics and reached a statistically significant level at 24 h compared with 0 and 6 h post infection (*P* = 0.015 and *P* = 0.018 respectively, Fig. 2c). In contrast, miR-22 expression in pBECs from asthmatics remained unchanged after infection. The different pattern of miR-22 expression in pBECs in asthmatics indicates a potential involvement of this miRNA in epithelial dysregulation after IAV H1N1 infection.

### miR-22 mimic suppresses and antagomir induces CD147 and HDAC4 mRNA expression

miR-22 expression is reported as a pro-differentiation miRNA [22] that regulates the expression of several targets including CD147 and HDAC4. Both targets have potential roles in airway remodeling and cell homeostasis [47–49]. To confirm CD147 and HDAC4 are targets of miR-22, minimally immortalized HBEC-6KT cells were transfected with miR-22 mimic (5 nM) or antagomir (5 nM) for 24 or 48 h, respectively. No morphological changes or signs of cell death was observed during transfection (Additional file 1: Figure S7A). Transfection was confirmed by assessment of miR-22 expression, using qPCR. Transfecting cells with miR-22 mimic resulted in significantly increased expression of miR-22 (Additional file 1: Figure S7B) while the antagomir inhibited the expression of miR-22 to levels below detection. Cells transfected with miR-22 mimic expressed lower levels of both CD147 and HDAC4 mRNA (Additional file 1: Figure S7C-D), whereas those transfected with miR-22 antagomir expressed higher expression of both genes (Additional file 1: Figure S7E-F). We observed no difference in protein expressions of CD147 and HDAC4 at these time points (data not shown). These data confirm that CD147 and HDAC4 genes are targets of miR-22.

**Fig. 3:**
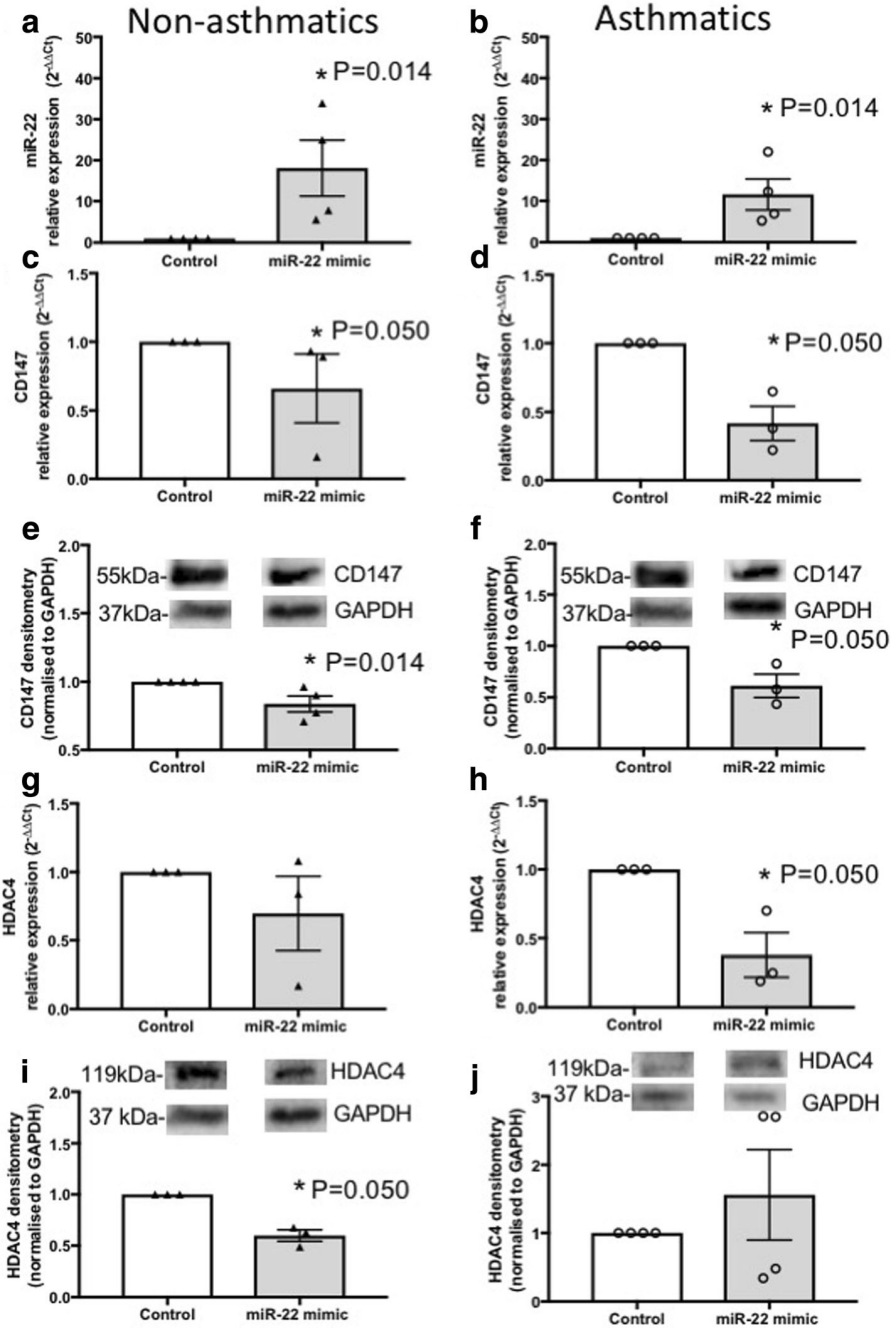
Effect of ectopic miR-22 mimic on CD147 and HDAC4 in pBECs of non-asthmatics and asthmatics. pBECs cultured as monolayers were transfected with miR-22 mimic (5 nM) for 24 or 48 h. **a**-**b** Transfection was confirmed by assessment of miR-22 expression, and the miR-22 mimic increased miR-22 expression after 24 h in both groups. **c**-**f** CD147 mRNA expression was reduced after 24 h in asthmatics and protein expression was suppressed after 48 h in both group. **g**-**j** HDAC4 mRNA expression was reduced in asthmatics after 24 h and protein expression was suppressed after 48 h in non-asthmatics. *P ≤ 0.05, using Mann-Whitney nonparametric test, *N* ≥ 3

To assess the functional role of miR-22, we transfected pBECs from non-asthmatics and asthmatics cultured as monolayers with miR-22 mimic (5 nM) for 24 h for mRNA and 48 h for protein level assessments. Transfection was confirmed by evaluating the induction of miR-22 expression in both non-asthmatics (*P* = 0.014) and asthmatics (*P* = 0.014), using qPCR (Fig. 3a-b). Over-expression of miR-22 suppressed CD147 mRNA and protein levels in pBECs from non-asthmatics (*P* = 0.050 and P = 0.014), and asthmatics (P = 0.050 and *P* = 0.050, Fig. 3c-f). Over-expression of miR-22 also suppressed HDAC4 protein levels (P = 0.050) in the pBECs of non-asthmatics and mRNA levels in asthmatics (*P* = 0.050, Fig. 3g-h).

### Expression of CD147 and HDAC4 in pBECs from non-asthmatics and asthmatics cultured at ALI

We next assessed the expression of CD147 and HDAC4 in pBECs grown at ALI following exposure to IAV H1N1.

**Fig. 4:**
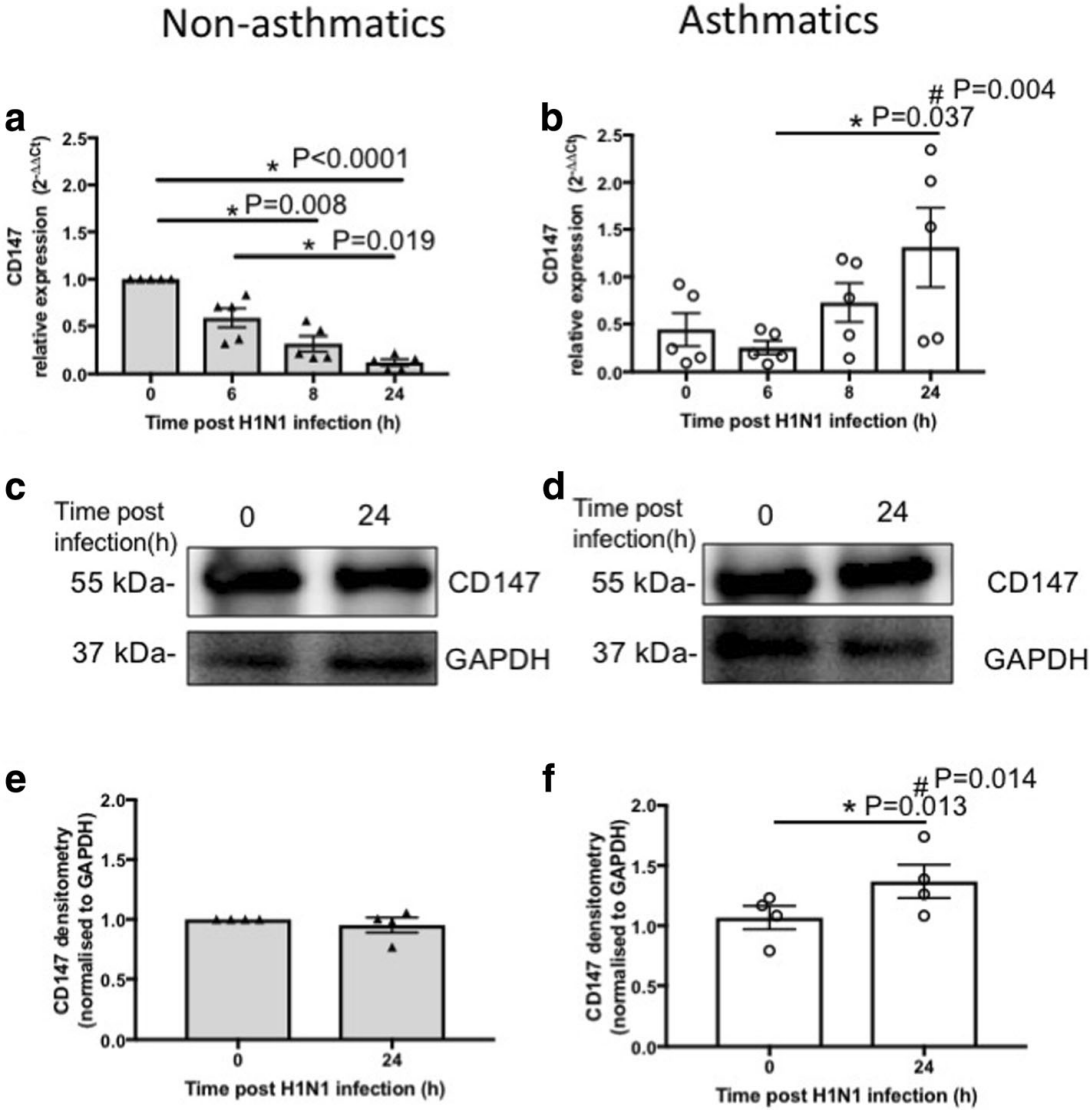
CD147 expression in pBECs from non-asthmatics and asthmatics cultured at ALI. Cells were infected with IAV H1N1 (MOI 5) and the expression of CD147 was assessed at different time points. **a** and (**b**) represent mRNA expression of CD147 at baseline (0 h) and after IAV H1N1 infection (6–24 h) in non-asthmatics and asthmatics, respectively. The cycle threshold (Ct) value was normalized to 18S gene (∆Ct) and data are presented relative to corresponding non-asthmatics at baseline. *P ≤ 0.05 intra-cohort, and #P ≤ 0.05 inter-cohort at 24 h, using the Kruskal Wallis multiple comparisons test and Mann-Whitney test, respectively, N=5. **c** and (**d**) are immunoblots representative of baseline and 24 h after infection in non-asthmatics and asthmatics, respectively. **e** and (**f**) represent densitometric quantification of immunoblots. Data are presented relative to corresponding non-asthmatic levels at baseline. *P ≤ 0.05 intra-cohort, and #P ≤ 0.05 inter-cohort at 24 h, using Mann-Whitney test, N=4

Basal (0 h) expression of CD147 at mRNA and protein levels in pBECs from non-asthmatics and asthmatics were comparable (Fig. 4). Following IAV H1N1 infection, expression of *CD147* decreased in pBECs of non-asthmatics over time, 0 vs 8 h (*P* = 0.008), 0 vs 24 h (*P* < 0.0001) and 6 vs 24 h (*P* = 0.019, Fig. 4a). In contrast, *CD147* expression increased and reached a significant level (*P* = 0.037) after 24 h compared with 6 h in pBEC from asthmatics (Fig. 4b). Consequently, expression of *CD147* was significantly higher (*P* = 0.004) in pBECs from asthmatics compared with non-asthmatics at 24 h post infection (Fig. 4a-b). CD147 protein expression was not affected by IAV H1N1 infection after 24 h in pBECs from non-asthmatics (Fig. 4c and e) but increased significantly (*P* = 0.013) in cells from asthmatics (Fig. 4d and f). Accordingly, CD147 protein levels were significantly higher (*P* = 0.014) in pBECs from asthmatics compared with non-asthmatics at 24 h after IAV H1N1 infection (Fig. 4e-f).

**Fig. 5:**
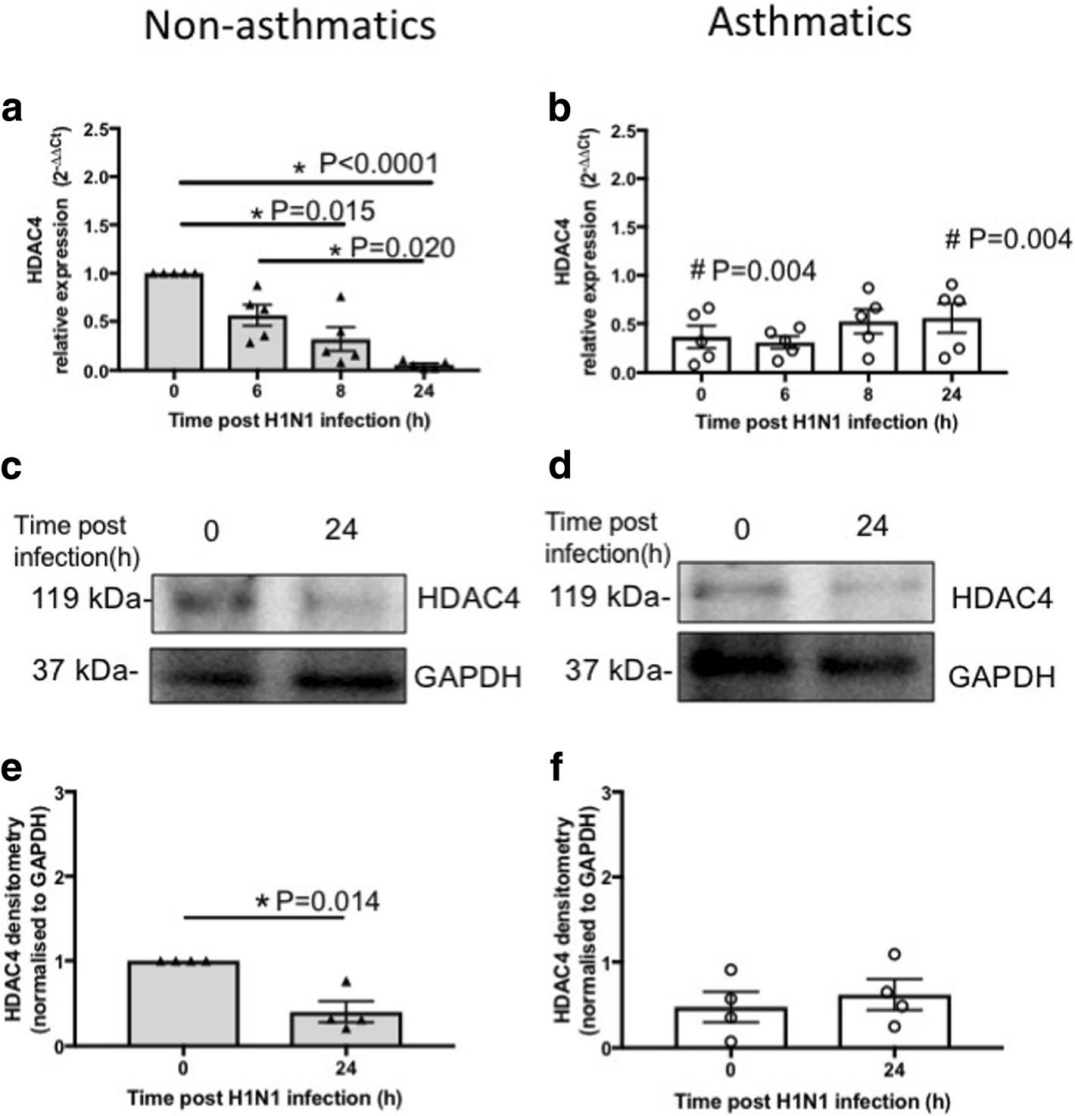
HDAC4 expression in pBECs from non-asthmatics and asthmatics cultured at ALI. Cells were infected with IAV H1N1 (MOI 5) and HDAC4 expression was assessed at different time points. **a** and (**b**) represent mRNA expressions of HDAC4 at baseline and after IAV H1N1 infection (6–24 h) in non-asthmatics and asthmatics, respectively. The cycle threshold (Ct) value was normalized to 18S gene (∆Ct) and data are presented relative to corresponding non-asthmatics at baseline. *P ≤ 0.05 intra-cohort, and #P ≤ 0.05 inter-cohort between corresponding time points, using the Kruskal Wallis multiple comparisons test and Mann-Whitney test, respectively, N=5. **c** and (**d**) are immunoblots representative of baseline and 24 h after infection in non-asthmatics and asthmatics, respectively. **e** and (**f**) represent densitometric quantification of immunoblots. Data are presented relative to corresponding non-asthmatic levels at baseline. *P ≤ 0.05, using Mann-Whitney test, N=4

We also observed that *HDAC4* expression was significantly lower (*P* = 0.004) in pBECs of asthmatics compared with non-asthmatics at baseline (Fig. 5a-b). Further, *HDAC4* expression was suppressed over time (0–24 h) significantly after IAV H1N1 infection in cells from non-asthmatics, 0 vs 8 h (*P* = 0.015), 0 vs 24 h (*P* < 0.0001) and 6 vs 24 h (*P* = 0.020, Fig. 5a) but remained unchanged in asthmatics (Fig. 5b). These led to lower levels (*P* = 0.004) of *HDAC4* in non-asthmatics compared with asthmatics at 24 h post infection (Fig. 5a-b). mRNA data match observations with protein levels as IAV H1N1 infection reduced HDAC4 protein levels significantly (*P* = 0.014) at 24 h compared with baseline in cells from non-asthmatics (Fig. 5c and e). However, HDAC4 protein expression was not affected by infection in cells from asthmatics (Fig. 5d and f).

These data show that CD147 and HDAC4 mRNA and protein levels differ after IAV H1N1 infection in cells from asthmatics compared with non-asthmatics.

### Expression and activity of MMP-9 in pBECs from non-asthmatics and asthmatics cultured at ALI

CD147 is known to induce matrix metalloproteinase (MMP) production (e.g. MMP-9) [48]. In addition, HDAC4 inhibition has been shown to prevent increases in MMP-9 and hence EMT in a mouse model of bleomycin-induced acute lung injury [50]. MMP-9 was reported to be increased in the airways of asthmatics and to play important multiple roles in tissue remodeling by degrading the extracellular matrix, and inducing inflammation [51, 52]. We therefore next assessed more functional aspects of miR-22 targets by measuring the expression and activity of MMP-9 in pBECs grown at ALI following IAV H1N1 infection.

**Fig. 6:**
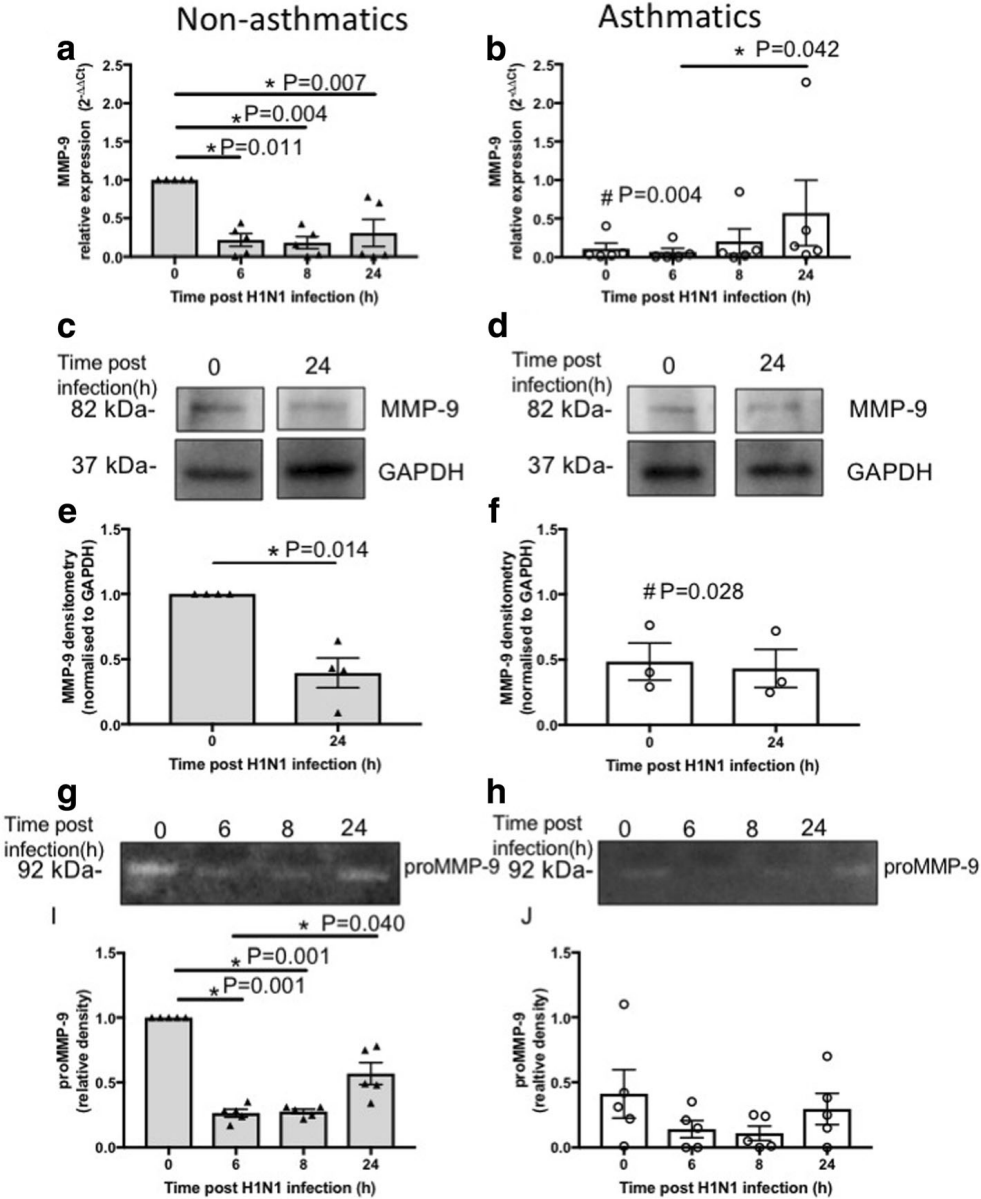
MMP-9 expression and activity in pBECs from non-asthmatics and asthmatics cultured at ALI. Cells were infected with IAV H1N1 (MOI 5) and MMP-9 expression was assessed at different time points. **a** and (**b**) mRNA expression of MMP-9 at baseline and after IAV H1N1 infection (6–24 h) in non-asthmatics and asthmatics. The cycle threshold (Ct) value was normalised to the 18S gene (∆Ct) and data are presented relative to corresponding non-asthmatics at baseline. *P ≤ 0.05 intra-cohort, and #*P* ≤ 0.05 inter-cohort between corresponding time points, using the Kruskal Wallis multiple comparisons test and Mann-Whitney test, respectively, *N* = 5. **c** and (**d**) are immunoblots representative of baseline and 24 h after infection in non-asthmatics and asthmatics, respectively. **e** and (**f**) Densitometric quantification of MMP-9 immunoblots. Data are presented relative to corresponding non-asthmatic levels at baseline. *P ≤ 0.05, using non-parametric test, *N* ≥ 4. **g** and (**h**) Representative zymograms after infection (0–24 h) in non-asthmatics and asthmatics. **i** and (**j**) Densitometric quantification of zymograms. Data are presented relative to corresponding non-asthmatic levels at baseline. *P ≤ 0.05 intra-cohort, and #*P* ≤ 0.05 inter-cohort between corresponding time points, using the Kruskal Wallis multiple comparisons test, *N* = 5

Basal levels of MMP-9 mRNA and protein in pBECs from non-asthmatics were significantly higher compared with asthmatics (P = 0.004 and *P* = 0.028, respectively, Fig. 6a-f). Following IAV H1N1 infection, mRNA expression of MMP-9 decreased in pBECs of non-asthmatics over time at 0 vs 6 h (*P* = 0.011), 0 vs 8 h (*P* = 0.004) and 0 vs 24 h (*P* = 0.007, Fig. 6a). Whereas, *MMP-9* expression increased and reached a significant level after 24 h compared with 6 h in pBEC from asthmatics (*P* = 0.042, Fig. 6b). MMP-9 protein levels reduced (*P* = 0.014) after IAV H1N1 infection (24 h) in pBECs from non-asthmatics (Fig. 6c and e) but were not affected by infection in cells from asthmatics (Fig. 6d and f). Using gelatin zymography we showed significant decreases in proMMP-9 in basal cell culture media after IAV H1N1 infection for 6 and 8 h (0 vs 6 h; *P* = 0.001 and 0 vs 8 h; *P* = 0.001) in pBECs of non-asthmatics (Fig. 6g and i). In contrast, infection had no effect on proMMP-9 activity in cells from asthmatics (Fig. 6h and j).

These data indicate that IAV H1N1 infection also has differential effects on MMP-9 expression in cells from asthmatics compared with non-asthmatics. Also, MMP-9 expression and activity are congruent with CD147 and HDAC4 expression in pBECs after infection particularly in cells from non-asthmatics.

### Expression of c-Myc and SP1 in pBECs from non-asthmatics and asthmatics cultured at ALI

In addition to miR-22, c-Myc and SP1 transcription factors are proposed to regulate CD147 gene transcription [53]. SP1 has also been reported to regulate HDAC4 [54]. Thus, we next sought to determine the effect of IAV H1N1 infection on the expression of these transcription factors.

**Fig. 7:**
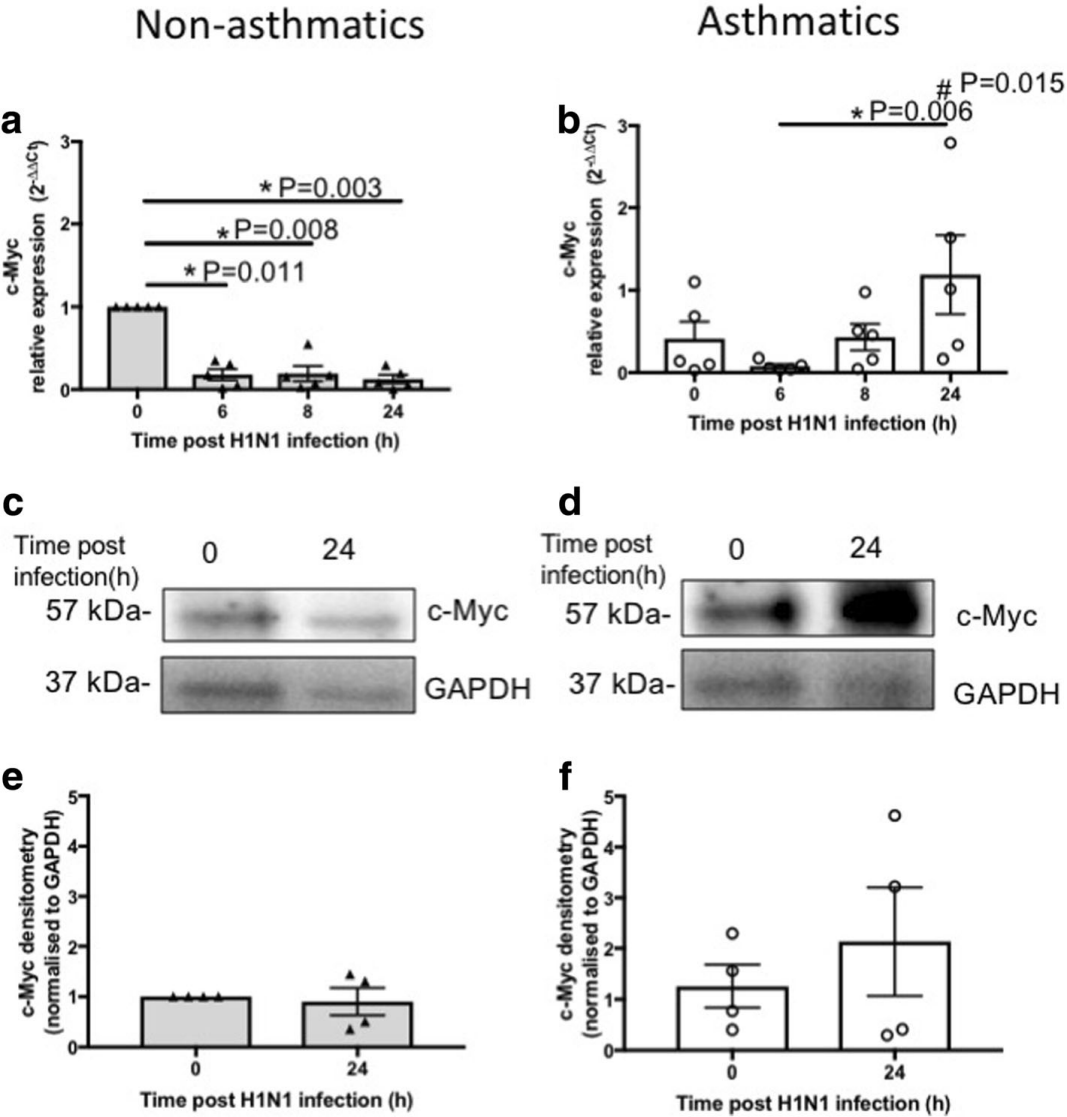
c-Myc expression in pBECs from non-asthmatics and asthmatics cultured at ALI. Cells were infected with IAV H1N1 (MOI 5) and the expression of c-Myc was assessed at different time points. **a** and (**b**) represent mRNA expression of c-Myc at baseline and after IAV H1N1 infection (6–24 h) in non-asthmatics and asthmatics, respectively. The cycle threshold (Ct) value was normalized to 18S gene (∆Ct) and data are presented relative to corresponding non-asthmatics at baseline. *P ≤ 0.05 intra-cohort, and #*P* ≤ 0.05 inter-cohort at 24 h, using the Kruskal Wallis multiple comparisons test and Mann-Whitney test, respectively, N=5. **c** and (**d**) are immunoblots representative of baseline and 24 h after infection in non-asthmatics and asthmatics, respectively. **e** and (**f**) represent densitometric quantification of immunoblots. Data are presented relative to corresponding non-asthmatic levels at baseline, *N* = 4

**Fig. 8:**
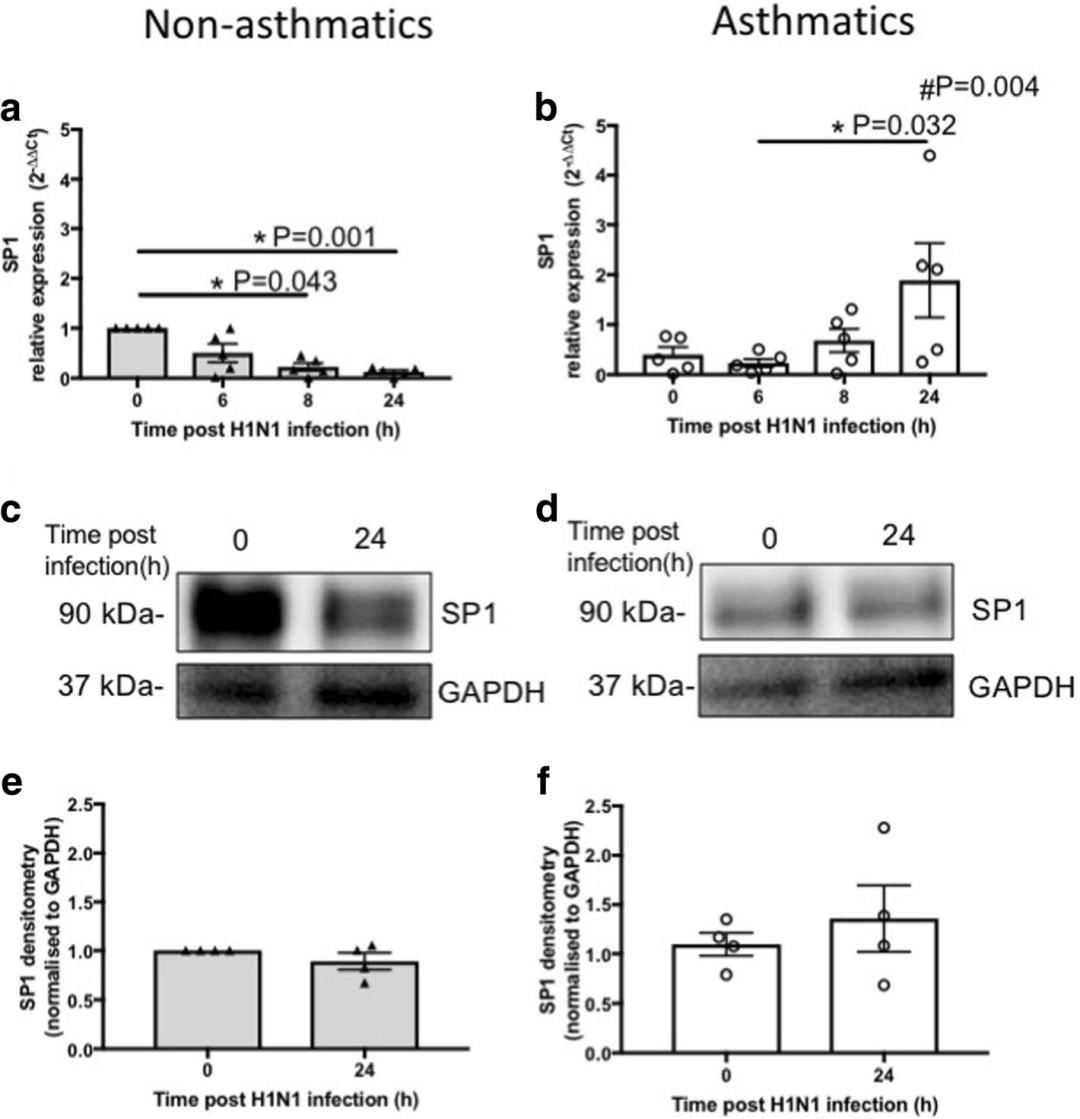
SP1 expression in pBECs from non-asthmatics and asthmatics cultured at ALI. Cells were infected with IAV H1N1 (MOI 5) and SP1 expression was assessed at different time points. **a** and (**b**) represent mRNA expression of SP1 at baseline and after IAV H1N1 infection (6–24 h) in non-asthmatics and asthmatics, respectively. The cycle threshold (Ct) value was normalized to 18S gene (∆Ct) and data are presented relative to corresponding non-asthmatics at baseline. *P ≤ 0.05 intra-cohort, and #*P* ≤ 0.05 inter-cohort at 24 h, using Kruskal Wallis multiple comparisons test and Mann-Whitney test, respectively, N=5. **c** and (**d**) are immunoblots representative of baseline and 24 h after infection in non-asthmatics and asthmatics, respectively. **e** and (**f**) represent densitometric quantification of immunoblots. Data are presented relative to corresponding non-asthmatic levels at baseline, N = 4

At baseline, no difference was observed in mRNA expression of c-Myc in pBECs of non-asthmatics compared to asthmatics (Fig. 7a-b). Following IAV H1N1 infection, expression of *c-Myc* reduced compared with baseline; 0 vs 6 h (*P* = 0.011), 0 vs 8 h (*P* = 0.008) and 0 vs 24 h (*P* = 0.003), and remained unchanged during infection (6–24 h) in pBECs from non-asthmatics (Fig. 7a) whereas its expression significantly increased (*P* = 0.006) in cells from asthmatics at 24 h compared with 6 h (Fig. 7b). Consequently, at 24 h post infection, mRNA expression of c-Myc was significantly higher (*P* = 0.015) in pBECs from asthmatics compared with non-asthmatics (Fig. 7a-b). At the protein level, IAV H1N1 infection did not affect c-Myc expression in cells from non-asthmatics (Fig. 7c and e), but increased c-Myc in asthmatics although not to a statistically significant level (Fig. 7d and f). We also observed comparable data with the assessment of SP1 expression at baseline and after IAV H1N1 infection (Fig. 8).

These data suggest that IAV H1N1 infection also has different effects on c-Myc and SP1 mRNA expression in cells from asthmatics compared with non-asthmatics, and that IAV H1N1 induced induction of *c-Myc* and *SP1* may increase CD147 expression in asthmatics.

## Discussion

We showed that under basal conditions, the pattern of miR-20a, − 132 and − 22 expression is similar in pBECs cultured in monolayer and differentiated ALI conditions, and are not affected by asthma. Our data support previous reports by Martinez-Nunez et al., who did not observe any changes in expression of these miRNAs in pBECs form non-asthmatics and asthmatics cultured as monolayers [38], as well as Williams et al., who also reported no significant differences in the expression of miRNAs in airway biopsies obtained from normal and mild asthmatic patients [39]. Moreover, IAV H1N1 infection did not affect the expression of these miRNAs when cells were cultured in submerged monolayers. In contrast, in cells cultured at the ALI, the kinetics of miR-22 expression was different after IAV H1N1 infection in cells from asthmatics compared with non-asthmatics. Specifically, miR-22 expression increased in pBECs of non-asthmatics but remained unchanged in asthmatics. We then found in non-asthmatics, the patterns of miR-22 expression were concomitantly opposite to CD147 gene, HDAC4 gene and protein, and their downstream MMP-9 gene and protein levels. In contrast, in pBECs from asthmatics, the expression of CD147 increased but miR-22, HDAC4 and MMP-9 expression remained unchanged after infection. To determine the mechanism responsible for IAV H1N1 induced induction of CD147 in cells from asthmatics, we further assessed the effect of infection on other regulatory factors of these candidate target genes. Our data indicate that IAV H1N1 infection induced c-Myc and SP1 gene expression in pBECs of asthmatics which may underpin the increased CD147 levels. In addition, we confirmed CD147 and HDAC4 are targets of miR-22. Comparing the effect of ectopic miR-22 mimic expression on its targets in pBECs of non-asthmatics with asthmatics showed that miR-22 overexpression suppressed CD147 in both cohorts whereas HDAC4 was suppressed at the protein level in non-asthmatics and mRNA level in asthmatics. These data indicate that miR-22 regulates CD147 and HDAC4 expression.

No differences were observed in morphology or physical properties of pBECs between non-asthmatics and asthmatics cultured as monolayers or at ALI. This is in agreement with previously reported data by Hackett et al., who showed that TEER was not different between pBECs from non-asthmatics and asthmatics [6]. While we did not assess different cell types or tight junction complexes, our TEER data were in contrast with Xiao et al., who showed tight junction and TEER was significantly lower in cultures from asthmatics than from non-asthmatics [55]. This discrepancy may be due to differences in sample numbers (5 vs 40). Furthermore, we observed similar levels of infection in pBECs from both groups at 24 h post infection which support data reported by Fujimoto et al. [56]. The virus titres in bronchoalveolar lavage fluid from a mouse model of bronchial asthma remained similar to control up to 2 days post IAV H1N1 infection and reached the highest level after 3 days of infection in an asthma model [56]. Despite these similarities, the different pattern of miR-22 expression in epithelial cells of adults with severe asthma may indicate the potential involvement of this miRNA in asthma pathogenesis. Our results highlight a novel mechanism of miR-22 mediated suppression of CD147 and HDAC4 in airway epithelial dysregulation that is associated with IAV H1N1 infection in severe asthma.

IAV causes significant morbidity and mortality in annual epidemics [14]. During the 2009 H1N1 swine flu pandemic in Australia and New Zealand, 33% of individuals who developed severe influenza and were admitted to hospital or ICU had asthma [57]. While vaccination is the major protective intervention against IAV H1N1 infection, its efficacy is challenged by new strains and therefore new vaccines have to be developed annually. Hence it is critical to understand the underlying mechanisms, such as miRNAs and their targets, responsible for severe responses of asthmatics to this infection to be able to provide more efficient interventions. While a number of miRNAs have been associated with abnormalities in asthmatic epithelium and disease progression, current asthma treatments (e.g. inhaled corticosteroids) show no major effect on them [39, 58, 59]. Hence, targeting miRNA abnormalities, using specific miRNA antagomirs or mimics, and restoring their normal functions may be an effective new intervention strategy.

The miR-20a is a member of miR-17-92 cluster which has essential roles in maintaining the structural homeostasis of the developing lung epithelium [21]. miR-20a is reported to be increased in the epithelium and mesenchyme of the embryonic compared with fully developed lung [21]. Our results, however, showed the same baseline level of miR-20a in pBECs from asthmatics compared with non-asthmatics cultured at both conditions. In addition, showed that miR-20a was unaffected by IAV H1N1 infection in pBECs form asthmatics compared with non-asthmatics in both culture conditions, which indicate this miRNA may not be responsive to the virus or its roles in epithelial cell homeostasis are more prominent during lung development.

Our data also showed that miR-132 is expressed at the same level in epithelial cells of asthmatics and non-asthmatics at baseline and after IAV H1N1 infection which was not dependent on culture conditions. Buggele et al., reported that Infection with IAV A/Udorn/72 H3N2 and A/WSN/33 H1N1 strains reported to increase miR-132 expression in the human lung epithelial cell lines A549 and BEAS-2B cells [26]. miR-132 has been shown to be induced in cells infected with Kaposi’s sarcoma-associated herpesvirus, and to target the histone acetyltransferase protein, p300, which is required for the production of IFNß [25]. However, we observed no effect of IAV H1N1 infection on miR-132 in epithelial cells from non-asthmatics or asthmatics in both culture conditions. These discrepancies could be due to the nature of cells; primary versus cell-line, or different strains of IAV A/Auckland/1/2009 H1N1 versus A/Udorn/72 H3N2 and A/WSN/33 H1N1.

Despite similar expression of miR-22 at baseline, our data indicate that after IAV H1N1 infection expression of this miRNA increased in pBECs of non-asthmatics whereas it was not altered in pBECs of asthmatics. miR-22 has been proposed to have tumor suppressive effects by targeting different genes responsible for cell proliferation [22, 23]. One of the targets of miR-22 is CD147 (EMMPRIN/basigin/HAb18G), which has a key role in tumor progression and metastasis [53]. CD147 induces the production of matrix metalloproteinases (MMP) such as MMP-9 that has important roles in airway remodeling and inflammation [48]. Patients with severe asthma are reported to have increased levels and activity of MMP-9 in their sputum compared with mild asthmatics and normal subjects [60]. Also, MMP-9 was shown to increase in airway epithelial cells in a mouse model of airway remodeling induced by ovalbumin challenge [52]. Furthermore, Jouneau et al., reported that epithelial cells are the major source for CD147 compared with alveolar and blood monocytes [48]. Another downstream target of miR-22 is HDAC4 [47]. HDAC4 is a key member of class IIa HDACs which plays an important role in tissue growth and development [61]. HDAC4 is known to be involved with TGF-ß1 induced epithelial-mesenchymal transition (EMT) in lung epithelial cells [49]. EMT is defined as a process that epithelial cells lose polarity and intercellular contacts, and adopt a mesenchymal phenotype. These changes are characterized by repression of epithelial genes, such as E-cadherin and ZO-1, and increased expression of mesenchymal proteins, including fibronectin, vimentin, and α-smooth muscle actin [33]. EMT can also lead to tissue remodeling and inflammation [49]. pBECs treated with TGF-ß1 also showed increased MMP-9 activity in cell culture supernatant [62]. There is evidence that epithelial cells of asthmatics are more prone to EMT [31]. Blocking HDAC4 reported to suppress TGF-ß1 associated inductions of α-smooth muscle actin, fibronectin and vimentin while restore TGF-ß1 related suppression of E-cadherin and TGF-ß1 induced migration of lung epithelial cells [49]. In addition, inhibiting HDAC4 suppressed MMP-9 elevation and hence EMT in a mouse model of bleomycin-induced acute lung injury [50]. Hence, MMP-9 is an important downstream factor of both CD147 and HDAC4 activity that is associated with tissue remodeling directly and via EMT processes.

Our data on MMP-9 expression are particularly interesting. To our knowledge there are no previous reports comparing MMP-9 expression in epithelial cells from asthmatics with non-asthmatics. MMP-9 mRNA and protein were expressed at lower levels in epithelial cells from asthmatics compared with non-asthmatics. However, it was previously shown that significant increases in MMP-9 in bronchoalveolar lavage positively correlated with levels of eosinophils and neutrophils in asthmatics compared with non-asthmatics [63]. These discrepancies may arise from the absence of other cells and hence lack of cell to cell communication in our model.

We showed that differential patterns of miR-22 expression may be a control factor to regulate CD147 and HDAC4 expression and their downstream MMP-9 expression and hence associated airway remodeling after IAV H1N1 infection in non-asthmatics. After infection, CD147, HDAC4 and MMP-9 mRNA levels reduced over time in association with increased miR-22 expression which prevents CD147 protein induction by maintaing its levels unchanged, and reduces HDAC4 and MMP-9 protein levels after infection in pBECs from non-asthmatics. However, these mechanisms differ in epithelial cells of asthmatics. Although miR-22 expression was not affected by IAV H1N1 in cells from asthmatics, CD147 increased at both gene and protein levels, potentially driven by infection induced *c-Myc* and *SP1* expression, whereas HDAC4 and their downstream target MMP-9 remained unchanged thus, potentially promoting airway remodeling. Overexpression of miR-22 in pBECs of asthmatics significantly suppressed CD147 expression. The miR-22 mimic may therefore offer some potential protective role by reversing CD147 over expression after IAV H1N1 infection in asthmatics to prevent further tissue remodeling.

Interestingly, IAV H1N1 infection showed no effect on miR-22 expression in cells in basal monolayers from asthmatics or non-asthmatics confirming that its effect is associated with differentiated cells. As for why the miR-22 pattern of expression differs in epithelial cells from asthmatics compared with non-asthmatics after infection, further molecular investigations are required and it is necessary to determine the effect of IAV on different cells types between two groups. Our data so far may imply that there is a recovery mechanism after IAV H1N1 infection in differentiated cells from non-asthmatics which leads to increase in miR-22 which is not present in asthmatics.

Due to the nature of exacerbations of asthma, investigating the epigenetic regulatory mechanisms within the airway epithelium is technically difficult, especially in response to specific exposures such as IAV. While the use of pseudostratified ALI epithelial cultures has several advantages over monolayer cultures, our study has limitations. One is that we only compared the pBECs at ALI culture of five subjects with asthma and five non-asthmatics. Regardless of the limitations in patient numbers, they were well-phenotyped. Another limitation is that we only investigated targeted miRNAs with different biological roles. However, miRNAs form networks and many of which have multiple binding partners and therefore affect multiple pathways. Thus, our next approach will be to assess global miRNAs and corresponding mRNA targets with high accuracy in our samples, using next gene sequencing technology. Furthermore, in our model, pBECs at passage 2 were differentiated over 23–25 days and during this period cell media was refreshed frequently. The effect of administered inhaled corticosteroids by patients was washed out during this process. However, to our knowledge there are no reports of the effects of common asthma therapeutics (e.g. corticosteroids) on miRNA expression in airway epithelial cells during IAV infection which warrants future investigations. Another limitation is that we did not assess which cell type was most infected with IAV H1N1 when cells were cultured at ALI conditions since we extracted miRNA, mRNA and protein from entire cell populations. However, this and how it affects miRNA expression in different cells (e.g. ciliated cells vs basal cells) will be interesting to determine. In addition, in our infection model, only MOI of 5 of IAV H1N1 was used. Future assessments on infection with IAV H1N1 at multiple MOIs (e.g. 0.5, 1, 5 and 10) will provide more evidence on regulation of miR-22 and its targets by this infection in pBECs. Also, administration of specific inhibitors of c-Myc or SP-1 during infection in cells from both groups, will determine the regulatory roles of these transcription factors on CD147 and HDAC4 in epithelial cells. Further, we assessed miRNA expression and their functions only in pBECs with or without infection, whereas co-culturing epithelial cells with relevant cells, e.g. neutrophils or macrophages which are also a source of MMP-9 in asthmatics [48], may affect miRNA expression and/or their targets in epithelial cells due to cell to cell crosstalk. Finally, our time course for IAV H1N1 infection was limited to 24 h.

## Conclusions

In conclusion, we demonstrate that in the absence of any stimulus, there is no difference in candidate miRNAs expressions between monolayer and ALI culture conditions. However, different cultures of epithelial cells respond differently to IAV H1N1 infection. We detect miR-22 and its targets CD147 and HDAC4 as potential responses to IAV H1N1 infection in epithelial cells with different patterns of expression in asthmatics compared with non-asthmatics. Our outcomes highlight a new response, which may potentiate airway remodeling associated with H1N1 infection in severe asthma.

## Additional file

Additional file 1: **Figure S1.** Monolayer cultures and generation of ALI-pBEC cultures from non-asthmatics and asthmatics. **Figure S2.** Viability of HBEC6-KT cell after IAV H1N1 infection at different MOIs. **Figure S3.** Responses of pBECs from non-asthmatics and asthmatics cultured as monolayers to IAV H1N1 infection. **Figure S4.** Responses of pBECs from non-asthmatics and asthmatics cultured at ALI to IAV H1N1 infection. **Figure S5.** RNU44 expression miRNA endogenous control in in pBECs from non-asthmatics and asthmatics cultured as monolayers and at ALI. **Figure S6.** UV-inactivated IAV H1N1 effects on levels of miRNA expression in pBECs from non-asthmatics and asthmatics. **Figure S7.** miR-22 mimic suppresses and antagomir increases CD147 and HDAC4 mRNA expression. (ZIP 499 kb)

## Abbreviations

ALI: Air-liquid-interface
EMT: Epithelial mesenchymal transition
GAPDH: Glyceraldehyde 3-phosphate dehydrogenase
HBEC6-KT: Minimally-immortalized bronchial epithelial cells
HDAC: Histone deacetylase
IAV: Influenza A virus
KSFM: Keratinocyte Serum-Free Media
MDCK: Madin-Darby canine kidney
miRNA: microRNA
MMP: Matrix metalloproteinases
MOI: Multiplicity of infection
pBEC: Primary bronchial epithelial cells
TEER: Transepithelial electrical resistance
