# Supplementary material for: Influenza A virus infection dysregulates the expression of microRNA-22 and its targets; CD147 and HDAC4, in epithelium of asthmatics
Source: Respir Res. 2018 Aug 2;19:145. doi: 10.1186/s12931-018-0851-7 (PMC6090696; doi:10.1186/s12931-018-0851-7)
Supplement: Supplementary file 1 — Figure S1. Monolayer cultures and generation of ALI-pBEC cultures from non-asthmatics and asthmatics. Figure S2. Viability of HBEC6-KT cell after IAV H1N1 infection at different MOIs. Figure S3. Responses of pBECs from non-asthmatics and asthmatics cultured as monolayers to IAV H1N1 infection. Figure S4. Responses of pBECs from non-asthmatics and asthmatics cultured at ALI to IAV H1N1 infection. Figure S5. RNU44 expression miRNA endogenous control in in pBECs from non-asthmatics and asthmatics cultured as monolayers and at ALI. Figure S6. UV-inactivated IAV H1N1 effects on levels of miRNA expression in pBECs from non-asthmatics and asthmatics. Figure S7. miR-22 mimic suppresses and antagomir increases CD147 and HDAC4 mRNA expression. (ZIP 499 kb) [file 12931_2018_851_MOESM1_ESM.zip › 12931_2018_851_MOESM1_ESM.pdf]

Influenza A virus infection dysregulates the expression of microRNA-22 and its targets; CD147 and HDAC4, in airway epithelium of asthmatics

Fatemeh Moheimani, Jorinke Koops, Teresa Williams, Andrew T. Reid, Philip M. Hansbro, Peter A. Wark, Darryl A. Knight

Online supplement

Figure S1. Monolayer cultures and generation of ALI-pBEC cultures from non-asthmatics and asthmatics. pBECs were cultured as submerged monolayers at passage 2 from (A) non-asthmatics and (B) asthmatics and showed no differences in morphology. For generation of ALI cultures, pBECs were cultured for 23–25 days (pre-infection). ALI cultures derived from (C) non-asthmatics and (D) asthmatics showed no morphological differences after hematoxylin and eosin staining (H&E). Scale bar is equal to 100 µm. Development of transepithelial electrical resistance (TEER) in cells from (E) non-asthamtics and (F) asthmatics, grown at ALI. *P≤ 0.05, using the Kruskal-Willis multiple comparisons test, N=5 and data are presented as mean ± SEM.

Figure S2. Viability of HBEC6-KT cell after IAV H1N1 infection at different MOIs. Cells cultured as monolayers were infected with IAV H1N1 (MOIs 0.5 to 20) or incubated with UV-incativated IAV H1N1 or media control for 24 h. Cell viability was determined by lactate dehydrogenase release assay. There was no significant effect of IAV H1N1 at MOIs 0.5, 1, 5 and 10 or equivalent UV-inactivated H1N1 on cell viability. IAV H1N1 infection with MOI 20 reduced cell viability significantly, using two-way analysis of variance with Bonferroni post-test, N=3 and Error bars show mean + SEM.

Figure S3. Responses of pBECs from non-asthmatics and asthmatics cultured as monolayers to IAV H1N1 infection. Cells were infected with IAV H1N1 (MOI 5). Photographs represent changes in appearance of MDCK cells incubated with different dilutions (4 to 64 times) of media (at 24 h post infection) for 72 h, N=5. The numbers of plaques were too dense to be counted by plaque assay. Scale bar is equal 100 μm.

Figure S4. Responses of pBECs from non-asthmatics and asthmatics cultured at ALI to IAV H1N1 infection. Cells were infected with IAV H1N1 (MOI 5). (A) Viral replication was measured by plaque assay after 24 h (N=5). (B) representative of crystal violet staining of the colonies at different dilutions of apical media after 24 h (10^5 to 10^9).

Figure S5. RNU44 expression miRNA endogenous control in in pBECs from non-asthmatics and asthmatics cultured as monolayers and at ALI. Cells were infected with IAV H1N1 (MOI 5). The expression of RNU44 was assessed at different time points of 0, 1, 4, 6 and 24 h, in cells from (A) non-asthmatics and (B) asthmatics cultured as moloayers. In cells cultured as ALI, RNU44 expression was assessed at time points of 0, 6, 8 and 24 h in (C) non-asthmatics and (D) asthmatics. RNU44 was expressed in pBECs and its expression remained unaffected by asthma or H1N1 IAV infection. Data are presented as the cycle threshold (Ct) value, N≥5 and error bars show mean + SEM.

Figure S6. UV-inactivated IAV H1N1 effects on levels of miRNA expression in pBECs from non-asthmatics and asthmatics. Cells were cultured as monolayers or ALI and incubated with UV-incativated IAV H1N1 in similar conditions as infection with IAV H1N1 (MOI 5). (A) miR-20a, (B) miR-132, and (C) miR-22 were expressed at similar level in cells incubated with UV-inactivated IAV H1N1 and media control, in submerged monolayer

cultures. Incubation with UV-inactivated IAV H1N1 showed similar expression of (D) miR-20a, (E) miR-132, and (F) miR-22 as media control, in ALI cultures. N≥3. The cycle threshold (Ct) value was normalized to that of the RNU44 (ΔCt). Data are presented relative to corresponding non-asthmatic levels at baseline.

Figure S7. miR-22 mimic suppresses and antagomir increases CD147 and HDAC4 mRNA expression. HBEC-6KT cells cultured as monolayers were transfected with miR-22 mimic (5 nM) or miR-22 antagomir (5 nM) for 24 or 48 h, respectively. (A) No phenotypic changes or cell death was observed. Scale bar is equal to 200 µm. (B) Transfection was confirmed by assessment of miR-22 expression, and the miR-22 mimic increased miR-22 expression. (C) *CD147* and (D) *HDAC4* expression was suppressed after transfection with miR-22 mimic for 24 h. (E) *CD147* and (F) *HDAC4* expression increased after transfection with miR-22 antagomir for 48 h. *P≤ 0.05, using nonparametric test, N=3
