# Supplementary figures and images for: Influenza A virus infection dysregulates the expression of microRNA-22 and its targets; CD147 and HDAC4, in epithelium of asthmatics
Source: Respir Res. 2018 Aug 2;19:145. doi: 10.1186/s12931-018-0851-7 (PMC6090696; doi:10.1186/s12931-018-0851-7)

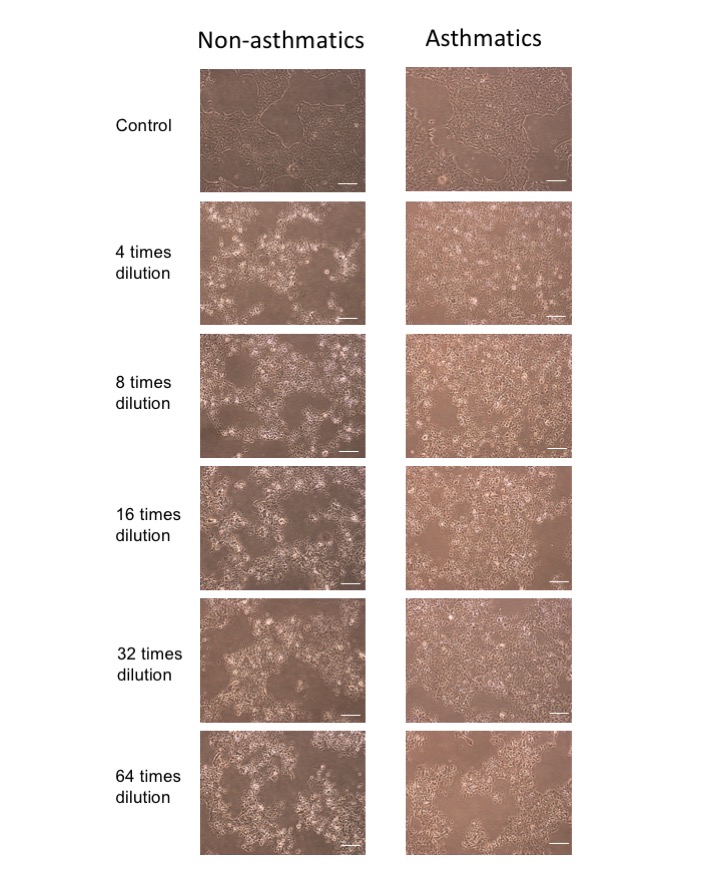

Supplement: Supplementary file 1 — Figure S1. Monolayer cultures and generation of ALI-pBEC cultures from non-asthmatics and asthmatics. Figure S2. Viability of HBEC6-KT cell after IAV H1N1 infection at different MOIs. Figure S3. Responses of pBECs from non-asthmatics and asthmatics cultured as monolayers to IAV H1N1 infection. Figure S4. Responses of pBECs from non-asthmatics and asthmatics cultured at ALI to IAV H1N1 infection. Figure S5. RNU44 expression miRNA endogenous control in in pBECs from non-asthmatics and asthmatics cultured as monolayers and at ALI. Figure S6. UV-inactivated IAV H1N1 effects on levels of miRNA expression in pBECs from non-asthmatics and asthmatics. Figure S7. miR-22 mimic suppresses and antagomir increases CD147 and HDAC4 mRNA expression. (ZIP 499 kb) [file 12931_2018_851_MOESM1_ESM.zip › Figure S3.jpeg]

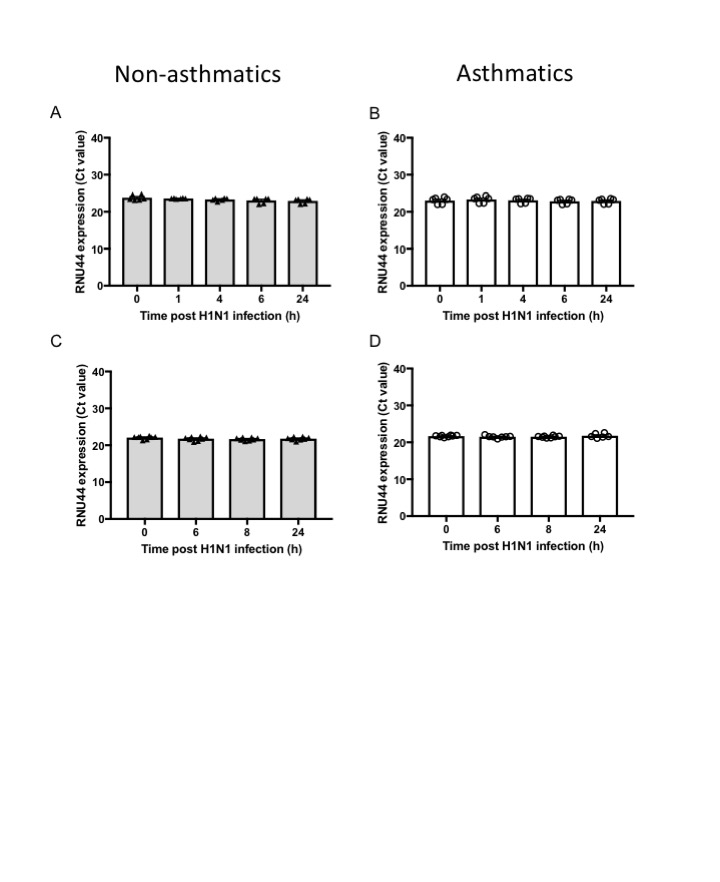

Supplement: Supplementary file 1 — Figure S1. Monolayer cultures and generation of ALI-pBEC cultures from non-asthmatics and asthmatics. Figure S2. Viability of HBEC6-KT cell after IAV H1N1 infection at different MOIs. Figure S3. Responses of pBECs from non-asthmatics and asthmatics cultured as monolayers to IAV H1N1 infection. Figure S4. Responses of pBECs from non-asthmatics and asthmatics cultured at ALI to IAV H1N1 infection. Figure S5. RNU44 expression miRNA endogenous control in in pBECs from non-asthmatics and asthmatics cultured as monolayers and at ALI. Figure S6. UV-inactivated IAV H1N1 effects on levels of miRNA expression in pBECs from non-asthmatics and asthmatics. Figure S7. miR-22 mimic suppresses and antagomir increases CD147 and HDAC4 mRNA expression. (ZIP 499 kb) [file 12931_2018_851_MOESM1_ESM.zip › Figure S5.jpeg]

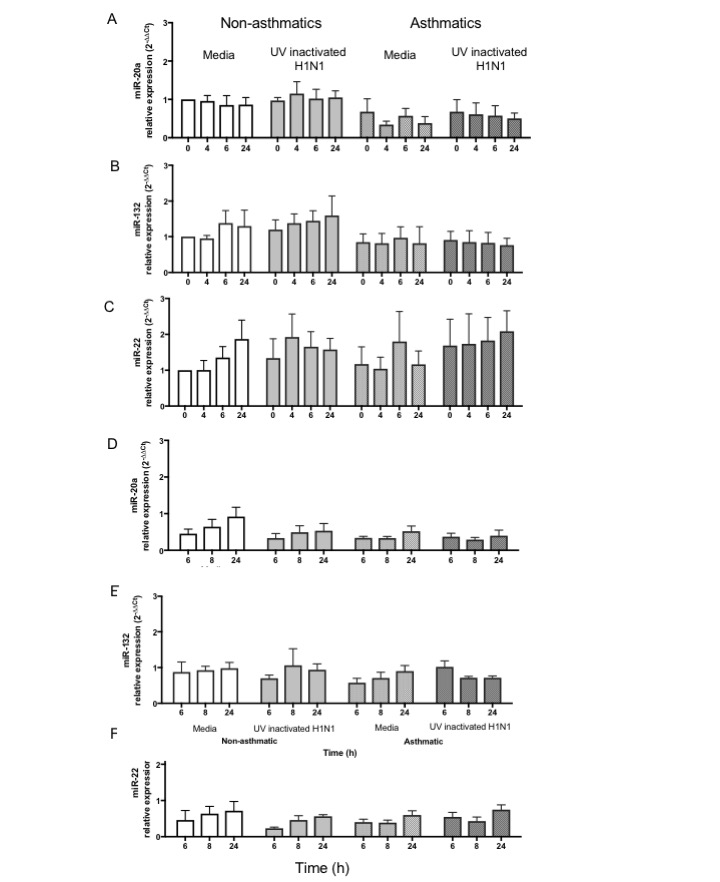

Supplement: Supplementary file 1 — Figure S1. Monolayer cultures and generation of ALI-pBEC cultures from non-asthmatics and asthmatics. Figure S2. Viability of HBEC6-KT cell after IAV H1N1 infection at different MOIs. Figure S3. Responses of pBECs from non-asthmatics and asthmatics cultured as monolayers to IAV H1N1 infection. Figure S4. Responses of pBECs from non-asthmatics and asthmatics cultured at ALI to IAV H1N1 infection. Figure S5. RNU44 expression miRNA endogenous control in in pBECs from non-asthmatics and asthmatics cultured as monolayers and at ALI. Figure S6. UV-inactivated IAV H1N1 effects on levels of miRNA expression in pBECs from non-asthmatics and asthmatics. Figure S7. miR-22 mimic suppresses and antagomir increases CD147 and HDAC4 mRNA expression. (ZIP 499 kb) [file 12931_2018_851_MOESM1_ESM.zip › Figure S6.jpeg]

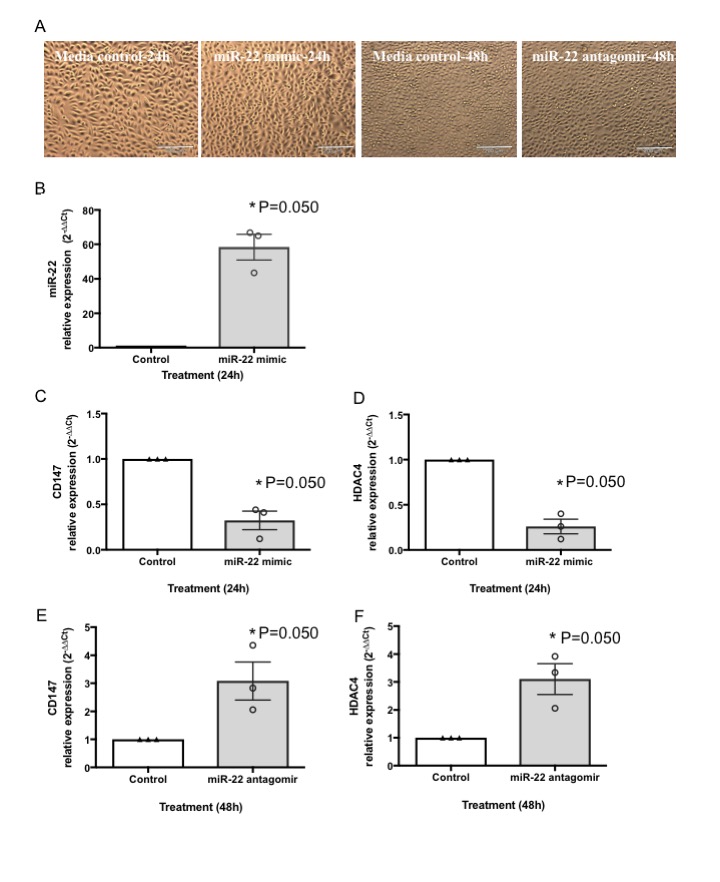

Supplement: Supplementary file 1 — Figure S1. Monolayer cultures and generation of ALI-pBEC cultures from non-asthmatics and asthmatics. Figure S2. Viability of HBEC6-KT cell after IAV H1N1 infection at different MOIs. Figure S3. Responses of pBECs from non-asthmatics and asthmatics cultured as monolayers to IAV H1N1 infection. Figure S4. Responses of pBECs from non-asthmatics and asthmatics cultured at ALI to IAV H1N1 infection. Figure S5. RNU44 expression miRNA endogenous control in in pBECs from non-asthmatics and asthmatics cultured as monolayers and at ALI. Figure S6. UV-inactivated IAV H1N1 effects on levels of miRNA expression in pBECs from non-asthmatics and asthmatics. Figure S7. miR-22 mimic suppresses and antagomir increases CD147 and HDAC4 mRNA expression. (ZIP 499 kb) [file 12931_2018_851_MOESM1_ESM.zip › Figure S7.jpeg]

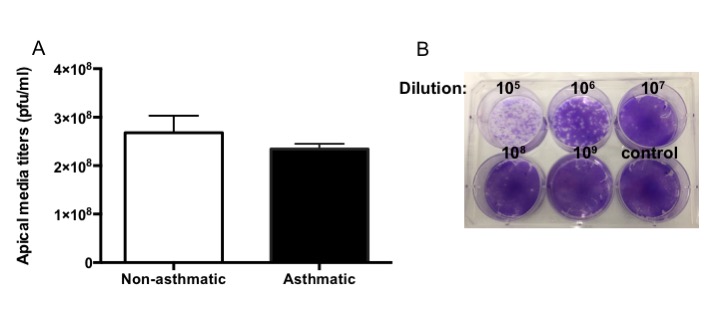

Supplement: Supplementary file 1 — Figure S1. Monolayer cultures and generation of ALI-pBEC cultures from non-asthmatics and asthmatics. Figure S2. Viability of HBEC6-KT cell after IAV H1N1 infection at different MOIs. Figure S3. Responses of pBECs from non-asthmatics and asthmatics cultured as monolayers to IAV H1N1 infection. Figure S4. Responses of pBECs from non-asthmatics and asthmatics cultured at ALI to IAV H1N1 infection. Figure S5. RNU44 expression miRNA endogenous control in in pBECs from non-asthmatics and asthmatics cultured as monolayers and at ALI. Figure S6. UV-inactivated IAV H1N1 effects on levels of miRNA expression in pBECs from non-asthmatics and asthmatics. Figure S7. miR-22 mimic suppresses and antagomir increases CD147 and HDAC4 mRNA expression. (ZIP 499 kb) [file 12931_2018_851_MOESM1_ESM.zip › Fig S4.jpeg]

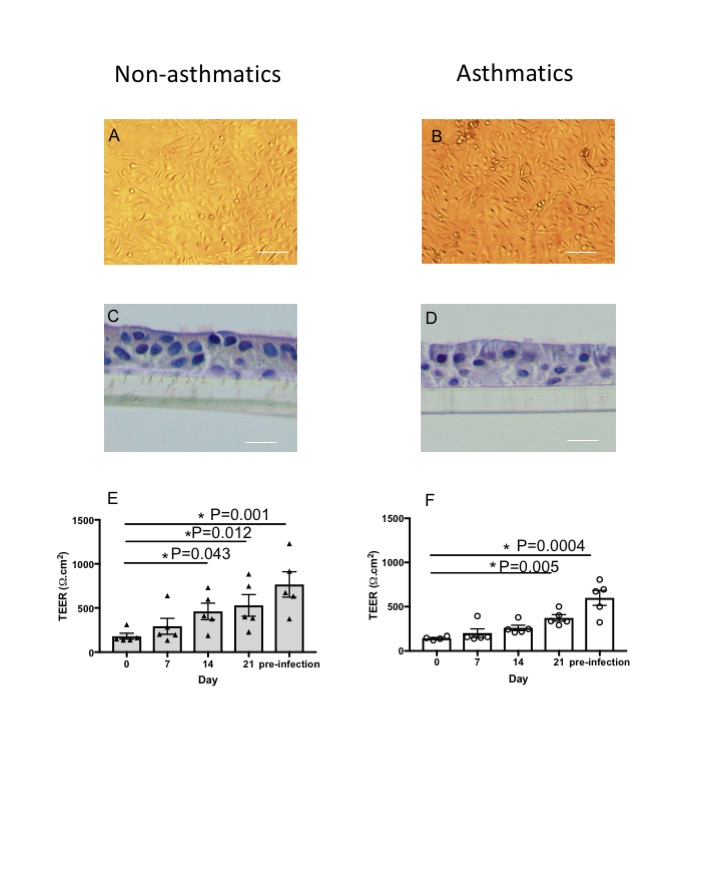

Supplement: Supplementary file 1 — Figure S1. Monolayer cultures and generation of ALI-pBEC cultures from non-asthmatics and asthmatics. Figure S2. Viability of HBEC6-KT cell after IAV H1N1 infection at different MOIs. Figure S3. Responses of pBECs from non-asthmatics and asthmatics cultured as monolayers to IAV H1N1 infection. Figure S4. Responses of pBECs from non-asthmatics and asthmatics cultured at ALI to IAV H1N1 infection. Figure S5. RNU44 expression miRNA endogenous control in in pBECs from non-asthmatics and asthmatics cultured as monolayers and at ALI. Figure S6. UV-inactivated IAV H1N1 effects on levels of miRNA expression in pBECs from non-asthmatics and asthmatics. Figure S7. miR-22 mimic suppresses and antagomir increases CD147 and HDAC4 mRNA expression. (ZIP 499 kb) [file 12931_2018_851_MOESM1_ESM.zip › Figure S1.jpeg]

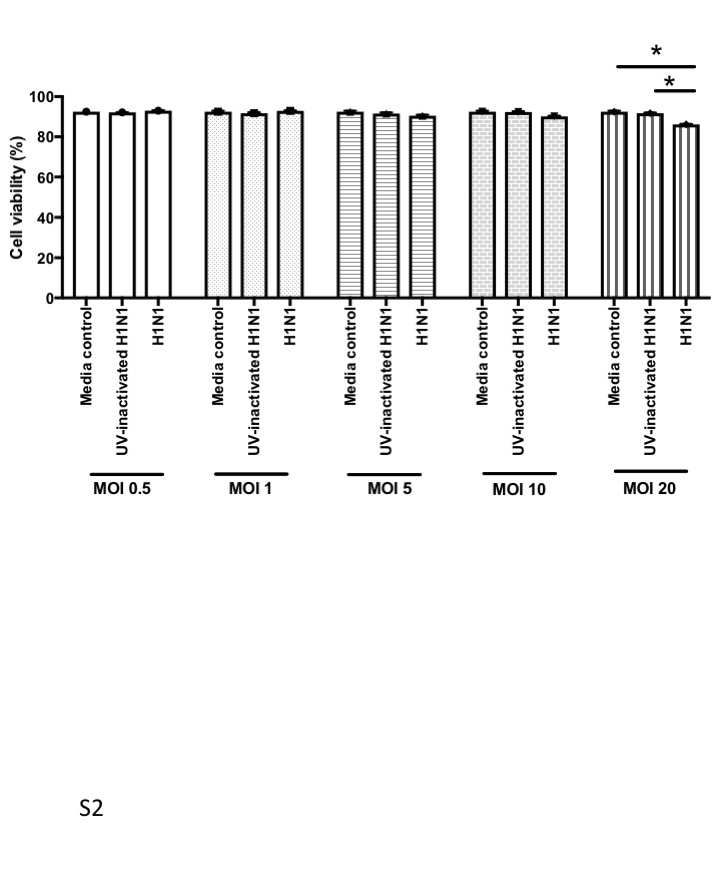

Supplement: Supplementary file 1 — Figure S1. Monolayer cultures and generation of ALI-pBEC cultures from non-asthmatics and asthmatics. Figure S2. Viability of HBEC6-KT cell after IAV H1N1 infection at different MOIs. Figure S3. Responses of pBECs from non-asthmatics and asthmatics cultured as monolayers to IAV H1N1 infection. Figure S4. Responses of pBECs from non-asthmatics and asthmatics cultured at ALI to IAV H1N1 infection. Figure S5. RNU44 expression miRNA endogenous control in in pBECs from non-asthmatics and asthmatics cultured as monolayers and at ALI. Figure S6. UV-inactivated IAV H1N1 effects on levels of miRNA expression in pBECs from non-asthmatics and asthmatics. Figure S7. miR-22 mimic suppresses and antagomir increases CD147 and HDAC4 mRNA expression. (ZIP 499 kb) [file 12931_2018_851_MOESM1_ESM.zip › Figure S2.jpeg]
